# Horseradish peroxidase assisted “signal-on” chemiluminescence aptasensor for sensitive detection of aflatoxin B1

**DOI:** 10.1039/d6ra00359a

**Published:** 2026-05-05

**Authors:** Linlin Sun, Fengyi Miao, Wenjuan Wan, Lingchen Wang, Chuan Dong

**Affiliations:** a Institute of Environmental Science, Shanxi University Taiyuan 030006 China sunlinlin@czmc.edu.cn; b Shanxi Higher Education Institutions of Science and Technology Innovation Plan Platform, Laboratory of Environmental Factors and Population Health, College of Public Health, Changzhi Medical College Changzhi 046000 China

## Abstract

Aflatoxin B1 (AFB1) analysis is crucial in food safety, environmental monitoring, and quality control. We developed a “signal-on” chemiluminescence aptasensor by using horseradish peroxidase (HRP)-labeled aptamer as affinity ligand, enabling the sensitive detection of AFB1. Complementary DNA (cDNA) of aptamer was immobilized on magnetic beads, and AFB1 in sample solution competed with cDNA coated on magnetic beads to bind to the HRP-labeled aptamer probe. The resulting AFB1-aptamer complexes were then magnetically separated and used for catalyzing substrate to produce chemiluminescence signals. Specifically, higher concentrations of AFB1 led to more AFB1-aptamer complexes being separated, thereby generating stronger chemiluminescence signals. Benefiting from the high catalytic activity of HRP for signal amplification and the advantages of chemiluminescence detection, this assay demonstrated high sensitivity to AFB1, with the detection limit of 0.01 nM. The dynamic range spanned from 0.01 nM to 500 nM, covering more than four orders of magnitude. The assay exhibited good selectivity and was successfully applied to detect AFB1 in diluted beer, liquor, and red wine samples. By leveraging the merits of aptamer, including high stability, good reproducibility, and facile chemical synthesis, this aptasensor holds great potential for practical AFB1 detection.

## Abstract 2

Aflatoxin B1 competes with cDNA to bind to HRP-labeled aptamer, which was used for catalyzing substrates to generate detection signals.

## Graphical abstract

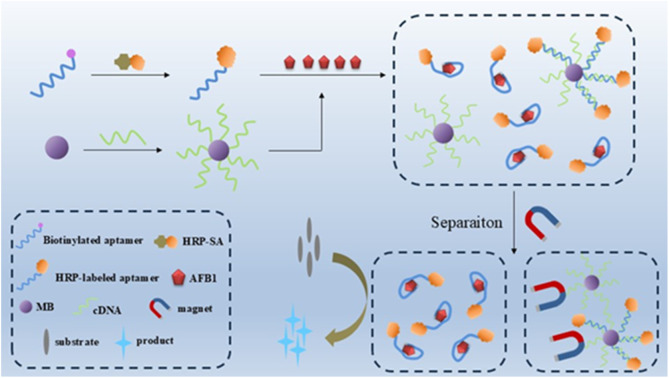

## 1. Introduction

Aflatoxin B1 (AFB1) is recognized as one of the most toxic mycotoxins, mainly produced by *Aspergillus flavus* and *Aspergillus parasiticus* that thrive in warm and humid conditions.^1,2^ The main AFB1 contaminated foods include staple grains (corn, peanuts, wheat), oilseeds (soybeans, sesame) and their processed products (peanut oil, cornmeal), and the contamination can occur at any stage of the food supply chain, from field harvesting to storage and processing.^1^ AFB1 poses severe health risks and is classified by the International Agency for Research on Cancer (IARC) as a Group 1 carcinogen, with long term exposure strongly linked to hepatocellular carcinoma.^3,4^ Acute toxicity of AFB1 exposure can cause liver damage, jaundice, and even death, while chronic exposure may lead to immune system suppression and stunted growth in children.^5,6^ Therefore, AFB1 detection plays a crucial role in preventing health risks and maintaining food safety. Conventionally, AFB1 detection still relies heavily on techniques such as high-performance liquid chromatography (HPLC) and enzyme-linked immuno-sorbent assay (ELISA).^7–9^ HPLC typically involves complex sample pretreatment, requires expensive instruments, and relies on skilled operators. ELISA depends on antibodies as recognition elements, and issues such as the instability and poor reproducibility of antibody limit its broader applicability. Therefore, there remains a demand to develop simple approaches capable of rapid, stable, and highly sensitive detection of AFB1.

Aptamers, short single-stranded nucleic acid sequences with high affinity and specificity to targets, have emerged as powerful molecular recognition elements.^10,11^ Compared to antibodies, aptamers offer significant advantages, like excellent stability, being easily chemically synthesized *in vitro* and allowing for easy modification with functional group.^12^ These properties have rendered aptamers highly valuable in biosensing and bioanalysis, where they play a pivotal role in detecting a variety of analytes, such as small molecules,^10,13^ proteins,^14^ cells,^15^ pathogenic microorganisms^16^ and *etc.*

Building on the high affinity of the aptamer screened by L.C. Le e*t al.* toward AFB1,^17^ various aptamer based sensors for AFB1 have been developed, including electrochemical,^18^ fluorescent,^19^ colorimetric^20^ and surface-enhanced Raman scattering (SERS) assays.^21^ Additionally, to meet the demand for trace AFB1 detection, different signal amplification strategies were introduced to aptamer based assays, like nanoparticles mediated signal amplification,^22,23^ enzyme based amplification,^19,24^ rolling circle amplification (RCA),^25,26^ strand displacement amplification^27,28^ and so on. Among these, horseradish peroxidase (HRP) is widely used as an enzyme label in immunoassays,^29^ biosensing,^24^ food safety detection^30^ and environmental monitoring^31^ owing to its high catalytic efficiency and broad substrate spectrum. Wang *et al.*^32^ reported a chemiluminescence sensor for AFB1 detection that utilizes RCA inside a pipet to enrich HRP (pipet-poly-HRP) and leverages the distinctive feature of the CRISPR/Cas12a system to cleave pipet-poly-HRP and release HRP for signal amplification. Yao *et al.*^33^ immobilized a short capture DNA probe (CP) on the surface of magnetic beads, which could partially hybridize with the aptamer to form a DNA duplex structure. Upon the addition of AFB1, the aptamer bound to AFB1, leaving the CP probe exposed on the magnetic beads after magnetic separation. Triggered by the sticky end of the CP probe, hybridization chain reaction (HCR) was carried out, through which HRP was indirectly immobilized on the surface of the magnetic beads. Finally, the quantitative detection of AFB1 was achieved by measuring the chemiluminescent signal generated by HRP-catalyzed reaction.

Herein, we developed an aptamer based chemiluminescence assay for AFB1 detection by using horseradish peroxidase (HRP) for signal amplification. HRP-labeled aptamer probe was prepared through the strong binding of HRP-streptavidin conjugate with biotinylated aptamer. Complementary DNA (cDNA) of aptamer was immobilized on magnetic beads. In the absence of AFB1 in sample solution, the aptamer hybridized with cDNA, resulting in the immobilization of HRP on the surface of magnetic beads and a low level of HRP remaining in solution. Upon addition of AFB1, the aptamer preferentially bound to AFB1 (due to its higher affinity toward AFB1) rather than hybridizing with the cDNA immobilized on magnetic beads, leading to an increased amount of HRP-labeled aptamer in solution. By means of an external magnetic, HRP-labeled aptamer probe bound to AFB1 was magnetically separated, which was used for catalyzing the chemiluminescent substrates to generate detectable signals. A higher content of AFB1 in sample correlates with an increased number of separated HRP-labeled aptamer probe, which in turn contributes to higher chemiluminescent signals. Due to the HRP assisted signal amplification and the features of chemiluminescence (low background, high sensitivity and short detection time), this assay achieved the sensitive detection of AFB1, with the detection limit of 0.01 nM, and showed the advantages of simple design, rapid detection, and high potential for practical application.

## 2. Experimental section

### 2.1 Materials and reagents

Aflatoxin B1 (AFB1) and other mycotoxins including ochratoxin A (OTA), fumonisin B1 (FB1), fumonisin B2 (FB2) and zearalenone (ZAE) were ordered from Pribolab (Singapore). Anti-AFB1 aptamer and complementary DNAs (cDNAs) were synthesized and purified by Sangon Biotech (Shanghai, China) and the sequences were listed in Table S1. These oligonucleotides each had one biotin labeled at the 3′ terminal with a tetra-ethyleneglycol (TEG) spacer. Horseradish peroxidase conjugated streptavidin (HRP-SA) was bought from Sangon Biotech (Shanghai, China). BM chemiluminescence ELISA substrate (POD) was obtained from Sigma (Germany). MagnaBindTM streptavidin were ordered from Thermo Scientific (USA). Liquor samples were all purchased from local market. The following buffer solutions were used. Assay buffer solution contained 10 mM HEPES, pH 7.5, 10 mM MgCl_2_, 50 mM NaCl and 0.1% Tween-20. Im-mobilization buffer solution for cDNA contained 20 mM tris–HCl, pH7.5, 2 M NaCl and 0.1% Tween-20. All solutions were prepared with ultrapure water obtained from Milli-Q Integral 15 System (Merck Millipore, USA). All other reagents were of analytical grade.

For chemiluminescence analysis, the substrate working solution was prepared by mixing 100 parts of reagent A (luminol and 4-iodophenol) with 1 part of reagent B (hydrogen peroxide), followed by stirring the mixture at room temperature for at least 15 min to activate the chemiluminescent signal. HRP can be activated in the presence of H_2_O_2_ and catalyzes the formation of radicals from 4-iodophenol and luminol, respectively. The two radicals react rapidly to generate excited-state 3-aminophthalate anion, which emits chemiluminescence upon relaxation to the ground state. As an enhancer, 4-iodophenol can significantly increase the intensity and stability of the luminescence. Finally, a plate reader (infinite 200, Tecan) and black 96-well plates ordered from Thermo Fisher Scientific (USA) were introduced to conduct chemiluminescence analysis. All AFB1 detection experiments were performed in triplicate (*n* = 3), and the results are presented as mean ± standard deviation.

### 2.2 Preparation of cDNA coated magnetic beads

The stock streptavidin coated magnetic beads (SA-MB) were firstly washed with immobilization buffer for three times and redispersed in immobilization buffer. Then equal volumes of SA-MB (10 mg mL^−1^) and biotinylated cDNA (each of 10 µM) were mixed and incubated at 25 °C for 1 h under mild shaking. After incubation, the beads were separated with an external magnetic field and were rinsed for three times with assay buffer. Finally, the cDNA-coated magnetic beads (cDNA-MB) were redispersed in assay buffer and stored at 4 °C for later use.

### 2.3 Chemiluminescence analysis of AFB1

For chemiluminescence analysis of AFB1, HRP-linked aptamer probe was firstly prepared by incubating HRP-SA conjugate and biotinylated aptamer (the ratio of 1 : 1) in assay buffer. Then the competing of AFB1 with cDNA to bind with HRP-linked aptamer probe was conducted by the following procedures at 4 °C. Different concentrations of AFB1 were incubated with HRP-linked aptamer probe (0.5 nM) in assay buffer for 10 min, and then 5 µL cDNA coated magnetic beads were added. After another incubation of 30 min, each sample was magnetically separated and the solution was par-allelly transferred into two parallel wells of microplate immediately. Subsequently, 100 µL chemiluminescence substrate working solution was added into each well. Following enzyme reaction of 3 min at room temperature, the chemiluminescence intensity was immediately measured by the plate reader.

### 2.4 Specificity test

To evaluate the specificity of this assay, some other mycotoxins, including OTA, FB1, FB2, ZAE and the mixture of these mycotoxins with AFB1 were tested by following the same procedures as AFB1 chemiluminescence detection in assay buffer. All of these mycotoxins were tested at 200 nM, while AFB1 was analyzed at 10 nM.

### 2.5 Detection of AFB1 in complex sample matrix

To test the feasibility of this assay for AFB1 detection in complex sample matrix, we performed the analysis of AFB1 in diluted beer, liquor and red wine samples. Firstly, the beer samples were taken into a beaker and sonicated for 2 h. After cooled at 4 °C for 30 min, the beer samples together with liquor and red wine samples were filtered with a 0.22 µm membrane and diluted by assay buffer. The beer and liquor samples were 20-fold diluted and the red wine samples were 50-fold diluted. By following, various concentrations of AFB1 were spiked in these diluted samples and analyzed with this aptamer based chemiluminescence assay.

## 3. Results and discussion

### 3.1 Assay principle and feasibility test

**Scheme 1:**
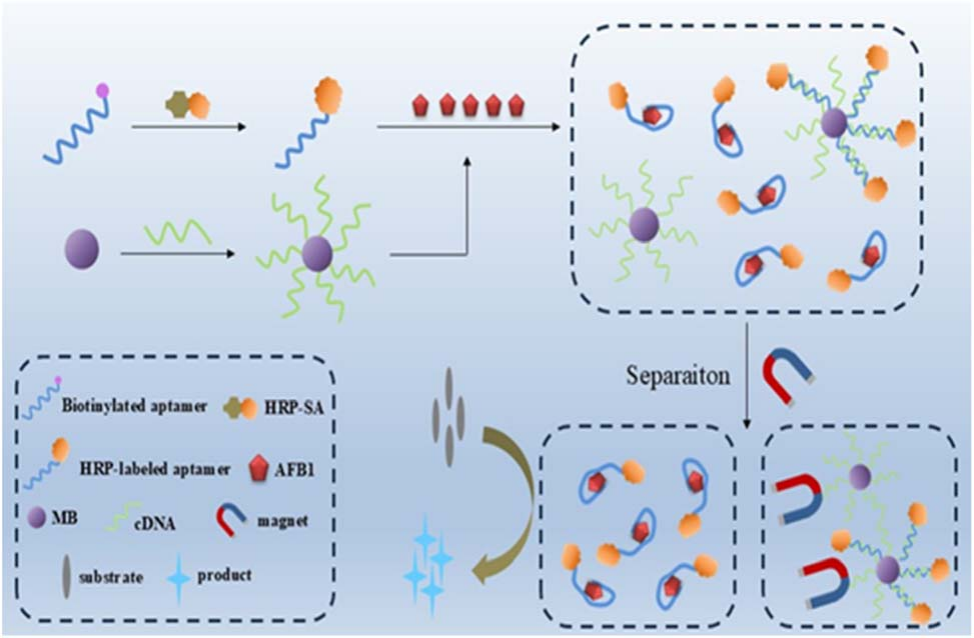
Schematic illustration of the chemiluminescence assay using horseradish peroxidase labeled aptamer for AFB1 detection.

Scheme 1 presents the design principle of this horseradish peroxidase assisted “signal-on” chemiluminescence aptasensor for AFB1 detection. Firstly, HRP-linked aptamer used for affinity probe is prepared by HRP-streptavidin (HRP-SA) covalent and biotinylated aptamer through the strong binding of streptavidin (SA) and biotin. The aptamer exhibits high binding affinity toward AFB1, with a dissociation constant (*K*_d_) of approximately 12 nM as reported in previous research.^34^ Consequently, free AFB1 in sample solution binds to the HRP-labeled aptamer probe to form an aptamer-AFB1 complex, resulting in less HRP being immobilized on the surface of magnetic beads *via* complementary hybridization between cDNA and the aptamer. Through magnetic separation, aptamer-AFB1 complex is isolated. With the increase of AFB1 in sample solution, more HRP-linked aptamer probes bind to AFB1, thus generating high chemiluminescence signals from the enzyme reaction products. AFB1 detection can be achieved by measuring the chemiluminescence signals with a plate reader, and the signal is expected to turn on with AFB1 addition.

**Fig. 1:**
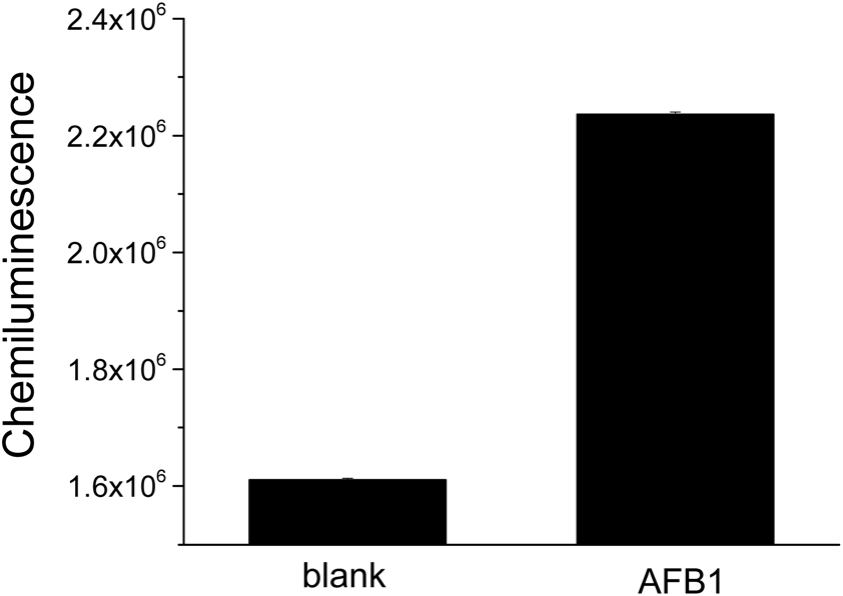
Feasibility test of this HRP-assisted “signal-on” chemiluminescence aptasensor for AFB1 detection. Blank sample and 10 nM AFB1 sample were tested.

We tested the feasibility of this chemiluminescence assay. As Fig. 1 shows, the chemiluminescence signal was low in blank sample, while AFB1 (10 nM) addition induced an obvious increase of the signal, enhancing it by approximately 1.4-fold. The results suggest that AFB1 competed with cDNA coated on magnetic beads for binding to the HRP-linked aptamer probe, leading to the formation of an aptamer-AFB1 complex. After magnetic separation, more HRP-linked aptamer probe remained in solution, resulting in a high chemiluminescence signal. Clearly, this HRP-labeled aptamer based chemiluminescence assay is feasible for AFB1 detection.

### 3.2 Optimization of assay conditions

To obtain better sensitivity for AFB1, we optimized several experimental conditions, including the length of cDNA coated on magnetic beads, the ratio of HRP-SA conjugate to biotinylated aptamer, the concentration of HRP-linked aptamer probe and the concentration of cations in assay buffer. The change of chemiluminescence signal caused by AFB1-binding was represented by CL/CL_0_ (%), which means the percentage of the signal value corresponding to AFB1 sample (CL) to that of blank sample (CL_0_).

**Fig. 2:**
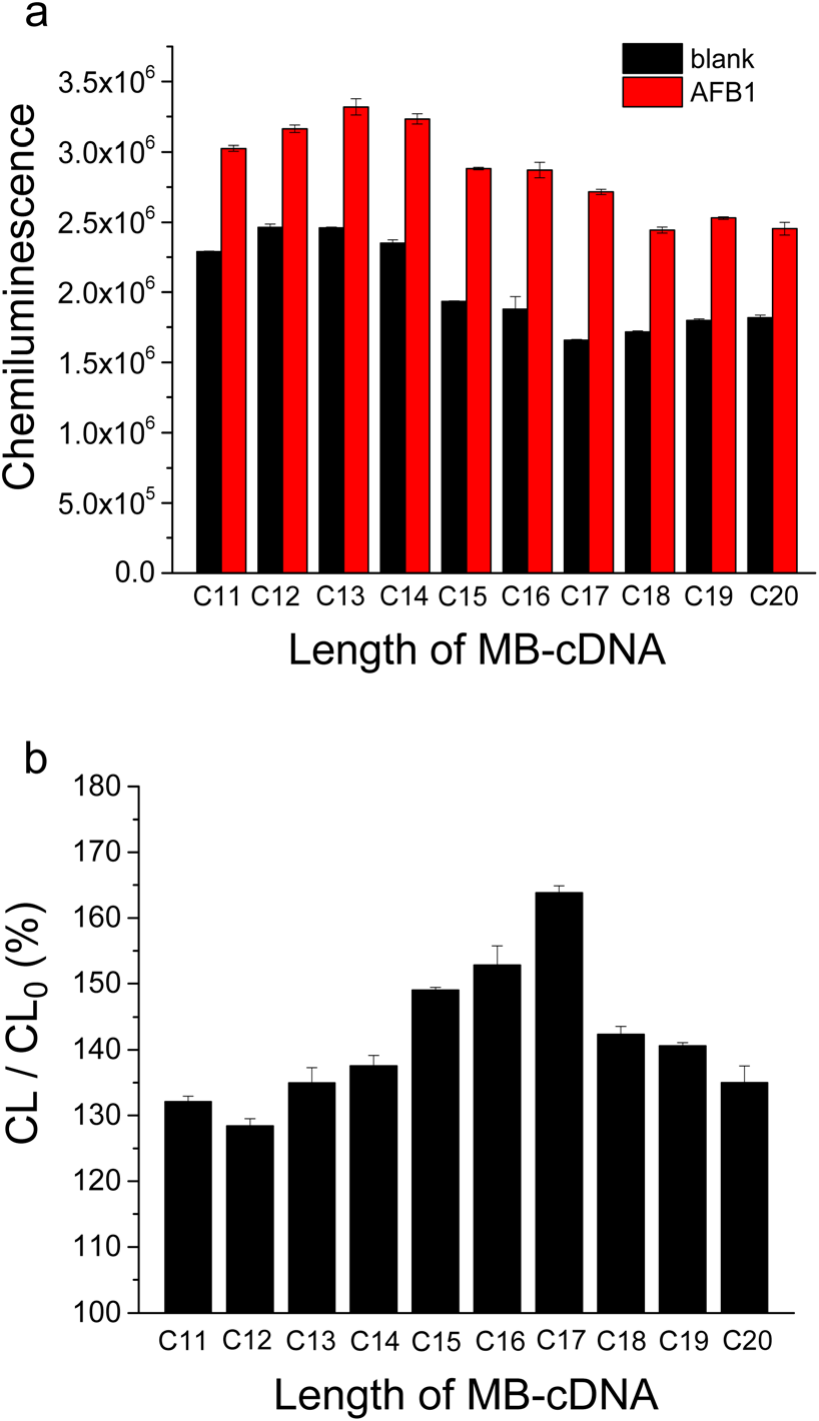
Effect of length of cDNA on AFB1 detection by this HRP assisted “signal-on” chemiluminescence aptasensor. (a) Chemiluminescence signals corresponding to blank sample and AFB1 sample (10 nM). (b) Change of chemiluminescence signal caused by AFB1-binding. CL/CL_0_ (%) means the percentage of the signal value corresponding to AFB1 sample (CL) to that of blank sample (CL_0_).

Firstly, the effect of cDNAs with lengths ranging from 11 to 20 nucleotides on AFB1 detection was investigated (Fig. 2). As shown in Fig. 2a, the chemiluminescence signals of both blank samples and AFB1 samples decreased slightly with an increase in the length of cDNA coated on magnetic beads. This phenomenon is attributed to the enhanced binding affinity between cDNAs with longer nucleotides and aptamer probes. Among the tested cDNAs, the 17-nucleotide cDNA (C17) showed the strongest response to AFB1 binding, with a 1.6-fold increase in chemiluminescence signal (Fig. 2b). Hence, C17 was selected as the optimal cDNA for subsequent AFB1 detection.

**Fig. 3:**
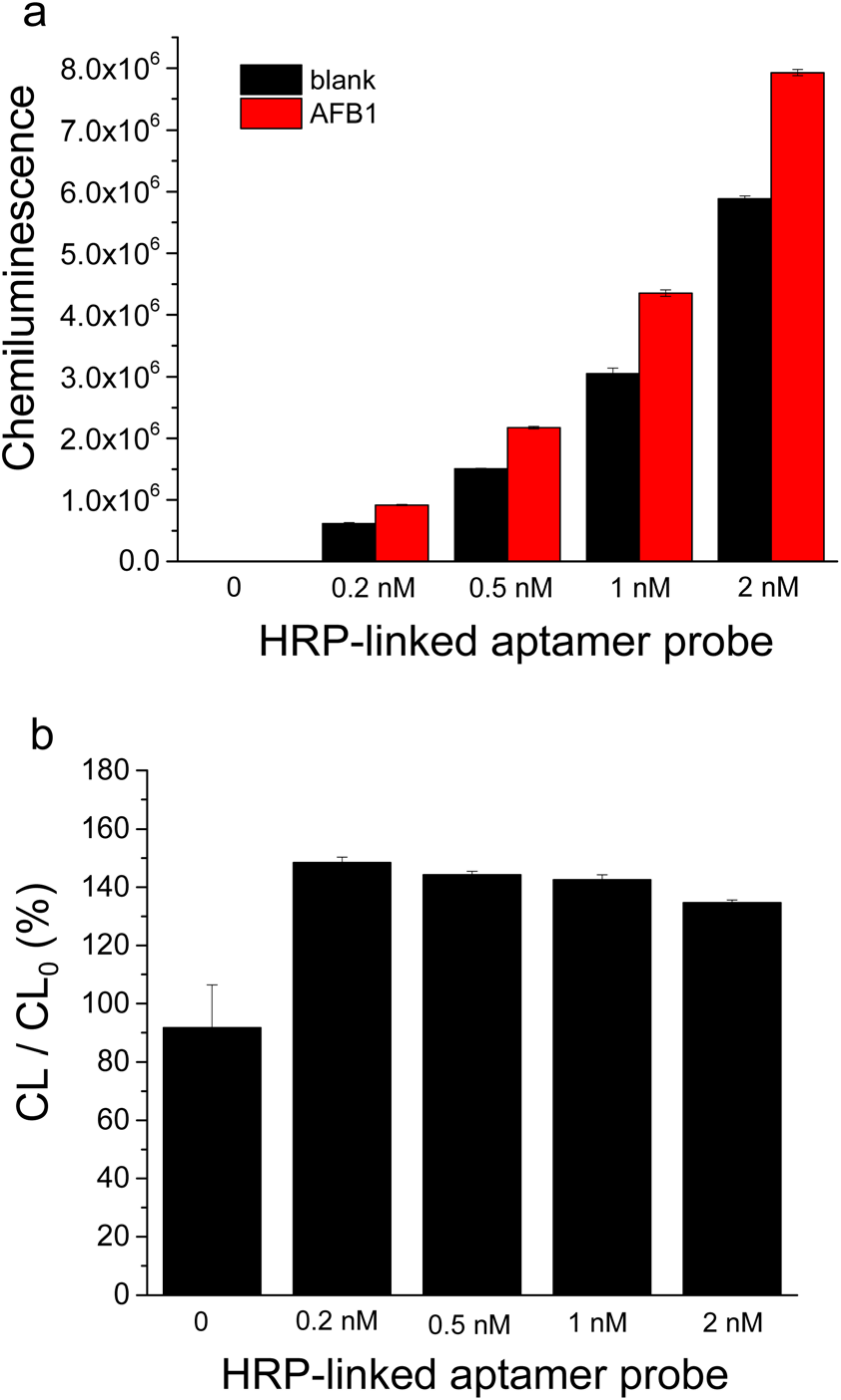
Effect of concentration of HRP-linked aptamer probe on AFB1 detection by this HRP assisted “signal-on” chemiluminescence aptasensor. (a) Chemiluminescence signals corresponding to blank sample and AFB1 sample (10 nM). (b) Change of chemiluminescence signal caused by AFB1-binding. CL/CL_0_ (%) means the percentage of the signal value corresponding to AFB1 sample (CL) to that of blank sample (CL_0_).

Given the critical role of HRP-linked aptamer probe in the competitive binding between AFB1 and cDNA, we further investigated the influence of HRP-labeled aptamer on AFB1 detection. The probe was first prepared by incubating HRP-streptavidin (HRP-SA) conjugate with biotinylated aptamer at different molar ratios. As shown in the SI (Fig. S1), the 1 : 1 molar ratio of HRP-SA to biotinylated aptamer yielded the maximal increase of chemiluminescence signal induced by 10 nM AFB1. Consequently, this 1 : 1 ratio was adopted for probe preparation in subsequent experiments. Subsequently, the effect of HRP-linked aptamer probe concentration on AFB1 detection was evaluated. As presented in Fig. 3, increasing the probe concentration led to gradual increases of chemiluminescence signals in both blank sample and AFB sample, accompanied by a slight reduction in the AFB1-induced signal change. To obtain optimal sensitivity and sufficient signal intensity, a final concentration of 0.5 nM HRP-linked aptamer probe was selected for AFB1 detection.

**Fig. 4:**
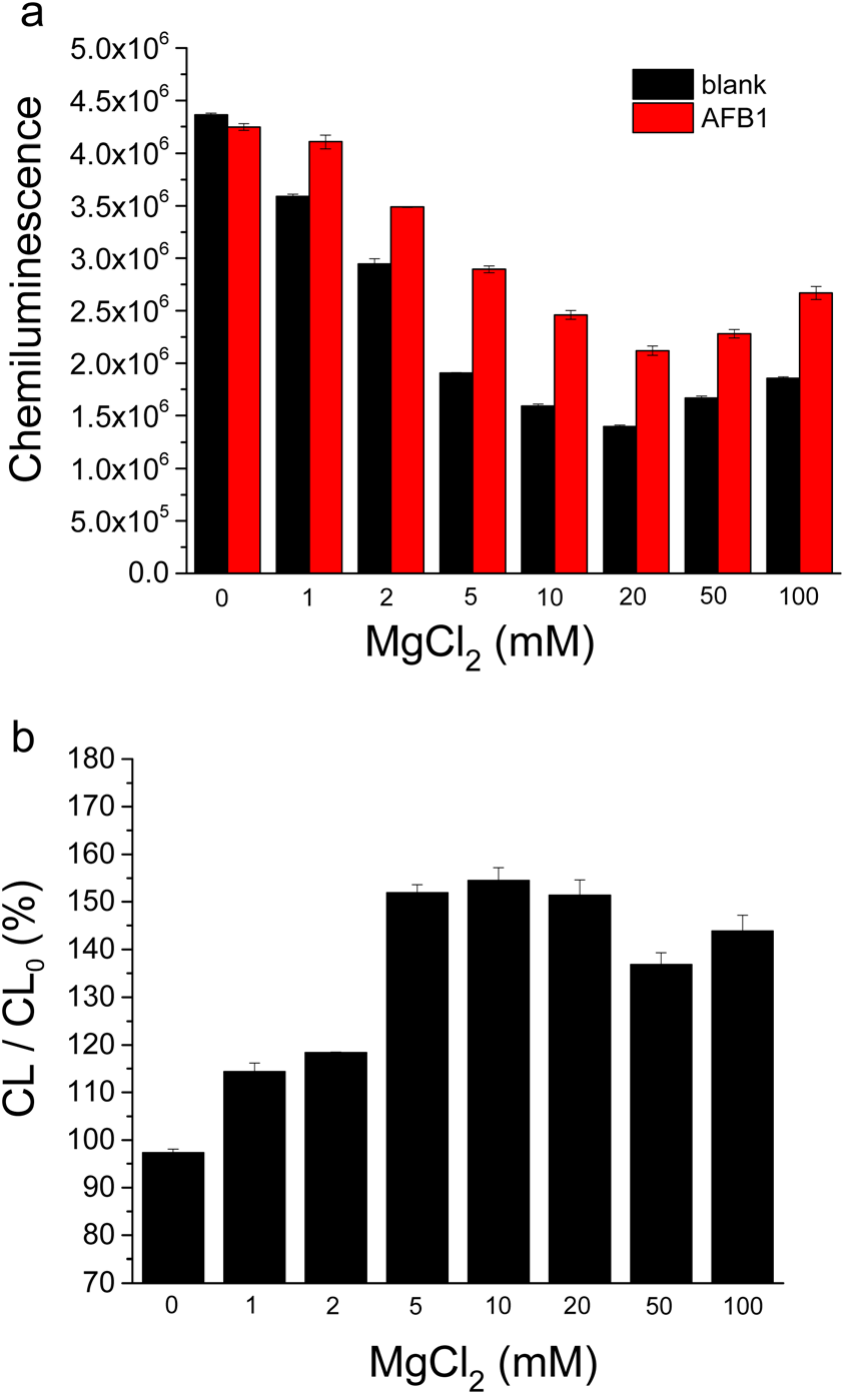
Effect of MgCl_2_ concentration on AFB1 detection by this HRP assisted “signal-on” chemiluminescence aptasensor. (a) Chemiluminescence signals corresponding to blank sample and AFB1 sample (10 nM). (b) Change of chemiluminescence signal caused by AFB1-binding. CL/CL_0_ (%) means the percentage of the signal value corresponding to AFB1 sample (CL) to that of blank sample (CL_0_). The buffer solution contained 10 mM HEPES (pH 7.5), 50 mM NaCl, 0.1% Tween-20 and various concentrations of MgCl_2_.

MgCl_2_ plays a crucial role in both the binding of AFB1 to aptamer, as well as the hybridization of cDNA with aptamer.^35,36^ We tested the influence of MgCl_2_ in assay buffer containing 10 mM HEPES (pH 7.5), 50 mM NaCl, 0.1% Tween-20 and various concentrations of MgCl_2_ (Fig. 4). As shown in Fig. 4a, the chemiluminescence signals of both blank sample and AFB1 sample decreased initially with increasing MgCl_2_ concentration, while a slight signal increase was observed when MgCl_2_ concentration exceeded 20 mM. Notably, the AFB1-induced chemiluminescence signal change increased significantly with increasing MgCl_2_ concentration and reached its maximum value at 10 mM MgCl_2_ (Fig. 4b). These results indicate that an appropriate concentration of MgCl_2_ promotes AFB1-aptamer binding, which is consistent with our previous findings.^35^ Given that 10 mM MgCl_2_ yielded the optimal AFB1-induced signal increase (about 1.5-fold), we added 10 mM MgCl_2_ in assay buffer for the following experiments.

We further evaluated the effect of NaCl concentration on assay performance by using assay buffers containing 10 mM HEPES (pH 7.5), 10 mM MgCl_2_, 0.1% Tween-20, and varying concentrations of NaCl (Fig. S2). At low NaCl concentrations, both blank sample and AFB sample exhibited weak chemiluminescence signals, accompanied by poor AFB1-induced signal variation. When NaCl concentration reached 50 mM, the maximal AFB1-induced signal change was observed. Consequently, the optimal assay buffer was determined as follows: 10 mM HEPES (pH 7.5), 10 mM MgCl_2_, 50 mM NaCl, and 0.1% Tween-20. Notably, the addition of Tween-20 was used to reduce non-specific adsorption.

### 3.3 Chemiluminescence assay for AFB1

**Fig. 5:**
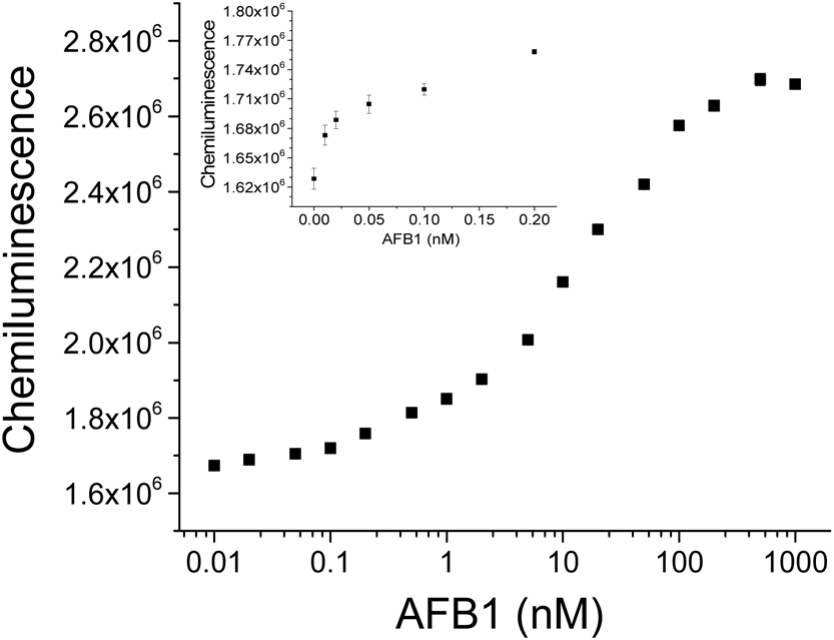
AFB1 detection using this HRP assisted “signal-on” chemiluminescence aptasensor. The inset shows the chemiluminescence signals corresponding to low concentrations of AFB1.

Under optimized conditions, we achieved successful AFB1 detection by using this HRP-labeled aptamer based chemiluminescence assay. As Fig. 5 shows, with the increase of AFB1 in solution, the chemiluminescence signals gradually increased. To show the details of signal changes corresponding to a wide concentration range of AFB1, a logarithmic scale was applied to AFB1 concentrations in Fig. 5. The dynamic detection range was from 0.01 nM to 500 nM, covering more than four orders of magnitude. The chemiluminescence signal was approximately 1.7-fold higher than that of the blank sample. Good linear fittings of chemiluminescence response to AFB1 concentrations ranging from 0.1 nM to 2 nM (*y* = 1.3624 × 10^5^ log *x* + 1.8534 × 10^6^, *R*^2^ = 0.9952) and AFB1 concentrations ranging from 2 nM to 200 nM (*y* = 3.7049 × 10^5^ log *x* + 1.7786 × 10^6^, *R*^2^ = 0.9960) were obtained by using OriginPro software, where *x* represents AFB1 concentration and *y* means chemiluminescence intensity. Based on a signal-to-noise ratio (S/N) of 3, the detection limit of AFB1 was determined to be 0.01 nM, which was lower than that of some reported aptasensors or immunoassays for AFB1 (summarized in Table 1). Compared to some other HRP-assisted chemiluminescent sensors for AFB1,^33,41,42^ this assay also showed comparable sensitivity, a wider detection range and a shorter assay time, which makes it suitable for scenarios with large concentration fluctuations in real samples and rapid screening. The high sensitivity was resulted from the signal amplification of HRP enzymatic reactions. Additionally, the introduce of chemiluminescence detection further enhanced the sensitivity and broadened the detection range. This aptamer-based assay, which capitalizes on the advantageous properties of aptamers, including high stability, excellent repeatability, and facile synthesis, offers a compelling alternative to traditional immunoassays.

**Table 1:** Comparation of some AFB1 detection methods

Recognition element	Method	Detection limit (nM)	Dynamic range (nM)	Ref.
Aptamer	Flower-like l-Cys-FeNiNPs nanozyme based colorimetric detection	117.02	384–6400	23
	Core-shell nanoparticles-based FRET assay	0.83	80–800	37
	One-pot isothermal assay combining rolling circle amplification and CRISPR/Cas12a	0.05	16–160	26
	Fluorescence aptasensor based on ligand-induced ssDNA displacement	0.70	4.8–588.2	27
	Nano-confined carbon/gold electrochemical assay	0.02	0.032–3.2 × 10^5^	38
	HRP- assisted chemiluminescence assay	0.01	0.01–500	This work
Antibody	MOF-encapsulated nanozyme-based chemiluminescence immunoassay	0.69	2.02–221.9	39
	Multicolour lateral flow immunoassay enabled by AIE nanoparticles	0.02	0.034–0.998	40

### 3.4 Specificity test

**Fig. 6:**
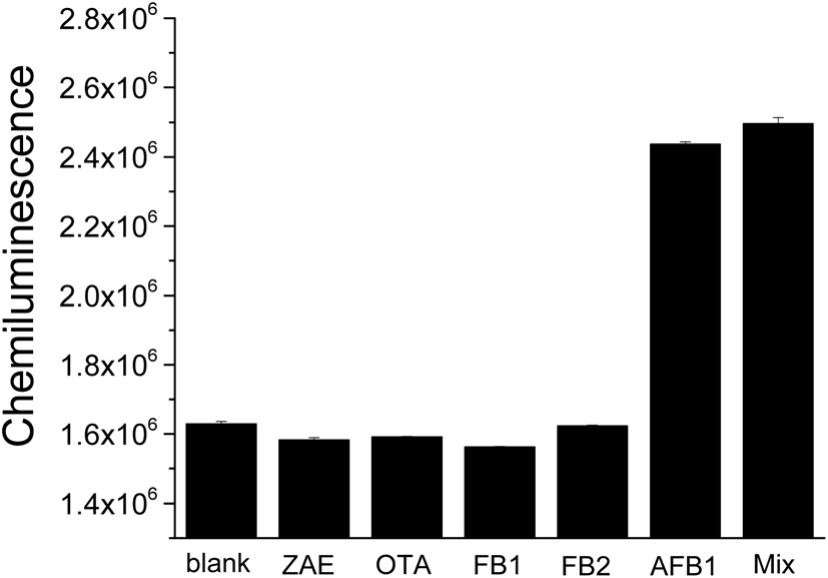
Specificity test of this HRP-assisted “signal-on” chemiluminescence aptasensor for AFB1 detection. Blank sample, other mycotoxins, including ZAE, OTA, FB1 and FB2 (each of 200 nM), AFB1 sample (10 nM), and the mixture containing ZAE, OTA, FB1, FB2 (each of 200 nM) and AFB1 (10 nM) were analyzed.

To test the specificity of this chemiluminescence assay for AFB1, several mycotoxins, including zearalenone (ZAE), ochratoxin A (OTA), fumonisin B1 (FB1) and fumonisin B2 (FB2) were tested. The chemiluminescence signals corresponding to these tested mycotoxin samples (200 nM) were significantly lower than that of AFB1 (10 nM) sample, and the mixture of these tested mycotoxins with AFB1 induced similar signal change with AFB1 sample (Fig. 6). The results indicate that this assay possesses good specificity toward AFB1 and the coexistence of these mycotoxins shows negligible influence on AFB1 detection.

### 3.5 Detection of AFB1 in complex sample matrices

**Fig. 7:**
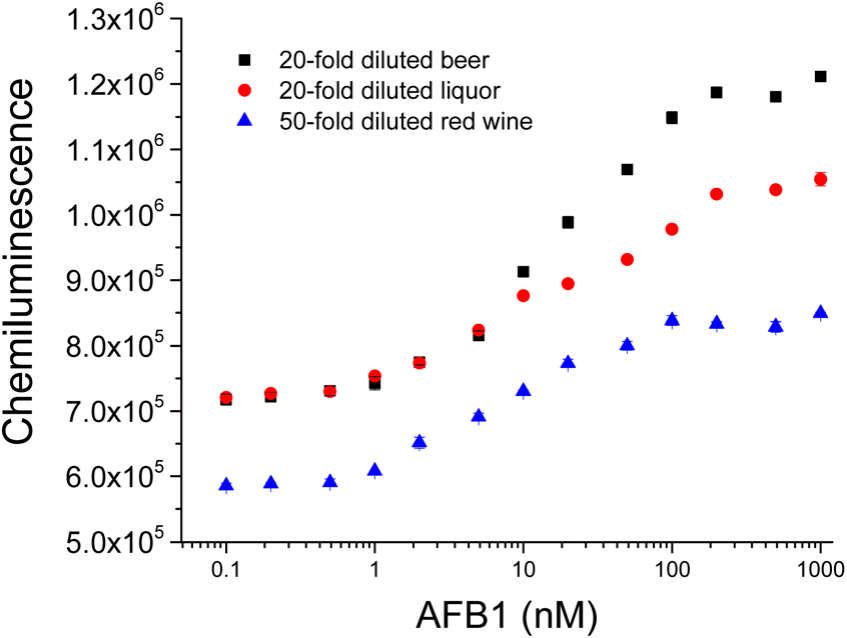
Detection of AFB1 spiked in 20-fold diluted beer, 20-fold diluted liquor and 50-fold diluted red wine by this HRP assisted “signal-on” chemiluminescence aptasensor.

To evaluate the performance of this assay for AFB1 detection in complex sample matrices, we detected AFB1 spiked in 20-fold diluted beer, 20-fold diluted liquor and 50-fold diluted red wine samples by using this HRP assisted “signal-on” chemiluminescence aptasensor. As shown in Fig. 7, the chemiluminescence signal increased gradually with increasing AFB1 concentration, indicating that our assay exhibited good responses to AFB1 in these texted sample matrices. Using a signal-to-noise ratio (S/N) of 3, the limits of detection (LOD) of AFB1 in 20-fold diluted beer, 20-fold diluted liq-uor and 50-fold diluted red wine samples were determined to be 1 nM and the dynamic range was from 1 nM to 100 nM. These results demonstrate that our chemiluminescence aptasensor is capable of detecting AFB1 in diluted beer, liquor and red wine samples.

## 4. Conclusions

In summary, we successfully constructed a “signal-on” chemiluminescence aptasensor by using HRP for signal amplification, which demonstrated excellent detection performance for AFB1. Under optimized conditions, this assay allowed sensitive detection of AFB1 as low as 0.01 nM, with a broad dynamic range of 0.01 nM to 500 nM. This assay exhibited good applicability in complex matrices including diluted beer liquor and red wine samples. This aptasensor integrates the merits of aptamer, such as high stability, good reproducibility, and ease of preparation, and shows the features of simple operation and rapid detection. It provides an effective approach for practical detection of AFB1.

## Author contributions

L. S. and L. W. designed the experiments, F. M., and W. W. conducted experiments. L. S. and F. M. wrote the manuscript. L. S., L. W. and C. D. revised the manuscript.

## Conflicts of interest

There are no conflicts to declare.

## Supplementary Material

RA-016-D6RA00359A-s001

## Data Availability

All data supporting the findings of this study are included within the article/supplementary information (SI). Additional details are available from the corresponding author upon reasonable request. Supplementary information: the sequences of DNA oligonucleotides used and the effect of the molar ratio of HRP-SA conjugate to biotinylated aptamer and NaCl concentration on AFB1 detections by this HRP assisted “signal-on” chemiluminescence aptasensor. See DOI: https://doi.org/10.1039/d6ra00359a.
