# Supplementary material for: Horseradish peroxidase assisted “signal-on” chemiluminescence aptasensor for sensitive detection of aflatoxin B1
Source: RSC Adv. 2026 May 5;16(25):23071–7. doi: 10.1039/d6ra00359a (PMC13142227; doi:10.1039/d6ra00359a)
Supplement: RA-016-D6RA00359A-s001 [file RA-016-D6RA00359A-s001.pdf]

Supplementary Information (SI) for RSC Advances.
This journal is © The Royal Society of Chemistry 2026

Supplementary Material

Horseradish Peroxidase Assisted "Signal-on" Chemiluminescence Aptasensor for Sensitive Detection of Aflatoxin B1

Linlin Sun[*a,b], Fengyi Miao[b], Wenjuan Wan[b], Lingchen Wang[a] and Chuan Dong[a]

a. Institute of Environmental Science, Shanxi University, Taiyuan 030006, China.

b. Shanxi Higher Education Institutions of Science and Technology Innovation Plan Platform, Laboratory of Environmental Factors and Population Health, College of Public Health, Changzhi Medical College, Changzhi 046000, China.

* Corresponding author

E-mail: sunlinlin@czmc.edu.cn

Table S1 Sequences of DNA oligonucleotides

Name	Sequences (5' to 3')
AF-Apt	TGGGCACGTGTTGTCTCTCTGTGTCTCGTGCCCTTTTTTT
C11-3BioTEG	ACACGTGCCCA
C12-3BioTEG	AACACGTGCCCA
C13-3BioTEG	CAACACGTGCCCA
C14-3BioTEG	ACAACACGTGCCCA
C15-3BioTEG	GACAACACGTGCCCA
C16-3BioTEG	AGACAACACGTGCCCA
C17-3BioTEG	GAGACAACACGTGCCCA
C18-3BioTEG	AGAGACAACACGTGCCCA
C19-3BioTEG	GAGAGACAACACGTGCCCA
C20-3BioTEG	AGAGAGACAACACGTGCCCA

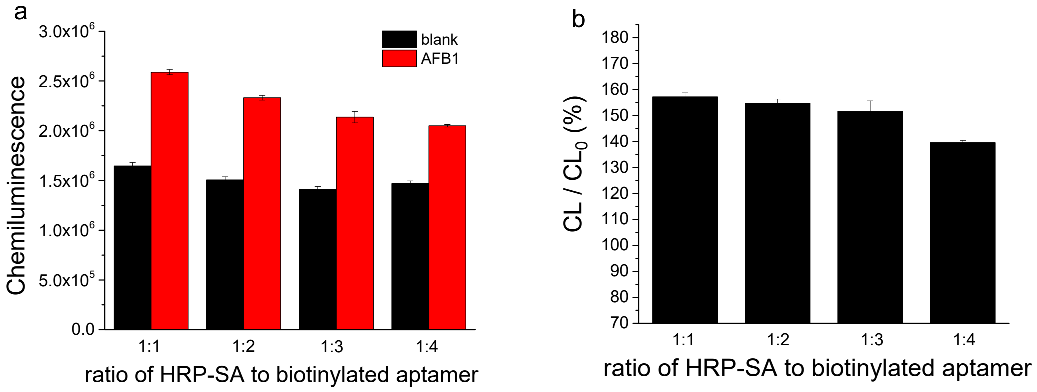

Fig.S1 Effect of the molar ratio of HRP-SA conjugate to biotinylated aptamer on AFB1 detection by this HRP assisted "signal-on" chemiluminescence aptasensor. (a) Chemiluminescence signals corresponding to blank sample and AFB1 sample (10 nM). (b) Change of chemiluminescence signal caused by AFB1-binding. CL/CL_0 (%) means the percentage of the signal value corresponding to AFB1 sample (CL) to that of blank sample (CL_0).

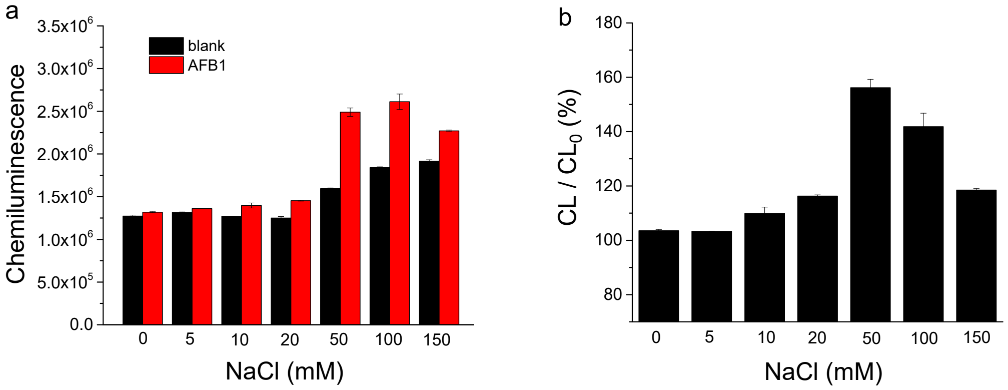

Fig.S2 Effect of NaCl concentration on AFB1 detection by this HRP assisted "signal-on" chemiluminescence aptasensor. (a) Chemiluminescence signals corresponding to blank sample and AFB1 sample (10 nM). (b) Change of chemiluminescence signal caused by AFB1-binding. CL/CL_0 (%) means the percentage of the signal value corresponding to AFB1 sample (CL) to that of blank sample (CL_0). The buffer solution contained 10 mM HEPES (pH 7.5), 10 mM $MgCl_2$, 0.1% Tween-20 and various concentrations of NaCl.
